# Bone Formation and Resorption Are Both Increased in Experimental Autoimmune Arthritis

**DOI:** 10.1371/journal.pone.0053034

**Published:** 2012-12-27

**Authors:** Kresten Krarup Keller, Jesper Skovhus Thomsen, Kristian Stengaard-Pedersen, Frederik Dagnæs-Hansen, Jens Randel Nyengaard, Ellen-Margrethe Hauge

**Affiliations:** 1 Department of Rheumatology, Aarhus University Hospital, Aarhus, Denmark; 2 Department of Biomedicine – Anatomy, Aarhus University, Aarhus, Denmark; 3 Stereology and Electron Microscopy Laboratory, Centre for Stochastic Geometry and Advanced Bioimaging, Aarhus University Hospital, Aarhus, Denmark; Faculté de médecine de Nantes, France

## Abstract

**Introduction:**

Arthritic bone loss in the joints of patients with rheumatoid arthritis is the result of a combination of osteoclastic bone resorption and osteoblastic bone formation. This process is not completely understood, and especially the importance of local inflammation needs further investigation. We evaluated how bone formation and bone resorption are altered in experimental autoimmune arthritis.

**Methods:**

Twenty-one female SKG mice were randomized to either an arthritis group or a control group. Tetracycline was used to identify mineralizing surfaces. After six weeks the right hind paws were embedded undecalcified in methylmethacrylate. The paws were cut exhaustively according to the principles of vertical sectioning and systematic sampling. 3D design-based methods were used to estimate the total number of osteoclasts, mineralizing surfaces, eroded surfaces, and osteoclast-covered bone surfaces. In addition the presence of adjacent inflammation was ascertained.

**Results:**

The total number of osteoclasts, mineralizing surfaces, eroded surfaces, and osteoclast covered surfaces were elevated in arthritic paws compared to normal paws. Mineralizing surfaces were elevated adjacent to as well as not adjacent to inflammation in arthritic mice compared to normal mice. In arthritic mice, eroded surfaces and osteoclast covered surfaces were larger on bone surfaces adjacent to inflammation than on bone surfaces without adjacent inflammation. However, we found no difference between mineralizing surfaces at bone surfaces with or without inflammation in arthritic mice.

**Conclusions:**

Inflammation induced an increase in resorptive bone surfaces as well as formative bone surfaces. The bone formative response may be more general, since formative bone surfaces were also increased when not associated with inflammation. Thus, the bone loss may be the result of a substantial local bone resorption, which cannot be compensated by the increased local bone formation. These findings may be valuable for the development of new osteoblast targeting drugs in RA.

## Introduction

The osteoclastic bone resorption in RA is relatively well understood [1], whereas only few studies investigating bone formation in RA is available [2]. Osteoclasts and osteoblasts are the central cells in bone turnover, and the function of these two cell types is coupled in many ways, e.g. through receptor activator of nuclear factor κB ligand (RANKL), RANK ligand (RANKL), and osteoprotegerin (OPG) [3]–[5]. How this coupling is disturbed in RA is not completely understood. However, it is well known that there is a net loss of bone locally in the affected joints [6] as well as a general osteoporosis [7]. Hence, the importance of the osteoclast and the osteoblast in arthritic bone loss should be further investigated.

Studies in humans indicate that repair of erosions does occur, and most often in patients with longstanding remission [8]. Moreover, MRI studies have documented that oedema in the bone marrow at diagnosis predicts poor radiographic prognosis years later [9]. However, histological studies investigating the importance of bone marrow inflammation in arthritis have shown ambiguous results [10], [11]. Thus, the impact of inflammatory tissue on adjacent bone formation is interesting and needs further investigation.

At present, most knowledge about local bone degradation originates from studies of cell cultures, whereas only few studies have addressed the importance of osteoclasts and osteoblasts using histological methods. Usually, bone histomorphometry is used, which is a two dimensional (2D) model-based method. This method can be problematic, because assumptions about shape, size, orientation, and distribution of the cells or tissue are made. In contrast, three dimensional (3D) stereological methods evaluate the tissue of interest in a design-based manner without assumptions about shape, size, orientation, and distribution. These new methods have proven useful in various other research areas [12]–[14].

In the present study we used the SKG mouse model of autoimmune polyarthritis described by Sakaguchi and coworkers [15]. The model is characterized by symmetric affection of small joints; elevation of Il-1, Il-6, TNF-α, Il-17, and rheumatoid factor; local and systemic bone loss; as well as inflammation of the skin, lungs, and blood vessels [15]–[20]. In a previous study we have demonstrated that the model is also characterized by an elevated number of osteoclasts and a higher proportion of osteoclast covered bone surfaces [21]. Thus, the SKG model shares many similarities with RA.

The purpose of the present study was to investigate how bone formation as well as bone resorption was altered in autoimmune arthritis and whether local inflammation had an impact on the adjacent bone formation and resorption.

## Materials and Methods

### Animals, Arthritis Induction, and Study Design

The study comprised 21 9–12-weeks-old female SKG mice, which were housed as previously described in detail [21]. The mice were randomized to an intraperitoneal (i.p.) injection with either 20 mg mannan (Sigma-Aldrich, USA) for induction of arthritis (n = 11) or placebo (PBS) for control (n = 10) [22]. Tetracycline (Sigma-Aldrich, USA) was administered i.p. at a dose of 30 mg/kg 8 days before termination of the study. Six weeks after arthritis induction the mice were anesthetized with isoflurane (Baxter, USA) and euthanized by cervical dislocation.

Arthritis score was performed twice weekly according to the SKG-scale [15]. Additionally, the width of the hind limb ankle joints was measured weekly with an electronic sliding caliper, and the mean width of the right and left ankle joint was calculated. Both measurements were performed by an observer blinded for the group distribution.

### Ethics Statement

The principles of laboratory animal care recommended by the US National Institute of Health were followed. The study was approved by the Danish Animal Experiments Inspectorate (permit number 2007/561-1317).

### Histological Preparation of Paws

**Figure 1:**
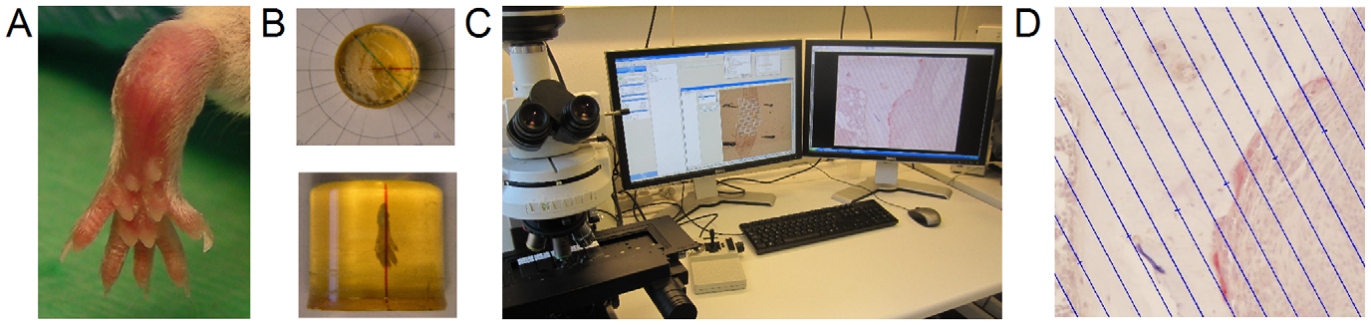
Application of stereological methods in arthritis research. (**A**) Right hinds paw from SKG mice were embedded in methyl methacrylate. (**B**) Paws were rotated randomly around the vertical axis prior to sectioning. (**C**) A stereology system including the newCAST software was used for quantification. (**D**) Surfaces were estimated by counting the number of intercept between a line grid and the tissue of interest.

After euthanasia the right hind paws were cut 0.5 cm above the ankle joint (Figure 1A), fixed in 70% ethanol, and embedded undecalcified in methylmethacrylate [23]. In general, shrinkage is minimal in plastic embedded tissue [24] and negligible in plastic embedded bone [25], and therefore, we did not correct for shrinkage.

Sections were cut using the principles of vertical sectioning [26]: The tissue blocks were rotated randomly around a vertical axis through the length axis of the paw (Figure 1B), and 7-µm-thick sections were cut exhaustively parallel to the rotation axis on a microtome (R. Jung GmbH, Heidelberg, Germany). Using the principle of systematic, uniformly random sampling approximately 10 levels each with 12 sections were generated for each paw [27].

The sections were stained for tartrate-resistant acid phosphatase (TRAP), stained with Masson-Goldner trichrome, or left unstained for fluorochrome analysis.

### Surface Estimation on Histological Sections

A microscope (Nikon ECLIPSE 80i, Tokyo, Japan) equipped with a motorized Proscan 11 stage (Prior, Cambridge, UK), a MT1201 microcator (Heidenhain, Traunreit, Germany), a fluorescence illuminator (Prior, Cambridge, UK), and a DP72 digital camera (Olympus, Tokyo, Japan) was connected to a PC with the stereological software newCAST (version 3.4.1.0, Visiopharm, Hørsholm, Denmark) (Figure 1C).

All parameters were evaluated in the tarsus at both the periosteal and the endosteal surface, and the presence of adjacent inflammatory tissue was evaluated. Inflammatory tissue was determined by the type and distribution of the cells observed. Quantification was performed with a line grid (Figure 1D) at a total magnification of ×457. In general, the aim was to achieve approximately 100 counts pr. animal for each parameter except for rare events (e.g. eroded surfaces in control mice), for which a low count was accepted. Aiming at a higher count would increase the workload, without changing the outcome.

Absolute osteoclast-covered bone surfaces (Oc.S) and absolute eroded bone surfaces (ES) were estimated on sections stained for TRAP. ES was defined as bone surfaces with breaks in the natural bone surface i.e. a scalloped surface indicating prior osteoclastic resorption at the surface. Absolute mineralizing surfaces (MS) were estimated on unstained sections. Absolute reference bone surfaces (BS) were estimated using Masson-Goldner trichrome stained sections. In arthritic mice, absolute reference bone surfaces adjacent to inflammatory or normal tissue was evaluated on sections stained for TRAP. Results are given as absolute values. When appropriate the results are given relative to the absolute bone surface. All samplings were performed by an observer blinded for the group distribution.

Surface parameters were estimated using the principles of the vertical sections design [26]. Previously, we have described the formula for estimating the surface parameters in detail [21]. Briefly, the area per length (*a*/*l*) was multiplied with the total number of intercepts in all sections (∑*I*) and the distance between the sections (*t*). In the present study we used a line grid with an *a*/*l* of 29.9 µm for estimation of Oc.S, ES, and MS, and an *a*/*l* of 74.6 µm for estimation of BS.

### Estimation of Osteoclast Number on Virtual Sections

Sections stained for TRAP were scanned in a high-resolution digital slide scanner generating virtual sections (NanoZoomer 2.0 series, Hamamatsu, Japan), which were subsequently analyzed using the newCAST system (version 3.6.5.0, Visiopharm, Denmark). We applied the Autodisector module in newCAST on the virtual slides thereby automatically generating physical disector pairs [28] at a total magnification of ×589 using a counting frame of 107912 µm^2^. Finally, an observer blinded for the group distribution counted osteoclasts and bridges using the disector pairs. Briefly, the principle of a disector is that two sections separated by a known distance (here 7 µm) are aligned and cell profiles are evaluated by a 2D counting frame. Cell profiles sampled by the 2D counting frame in the reference section, but not in the look-up section, are counted. A bridge is defined as when a cell profile in one section turns into two cell profiles in the other section of the disector [21].

The absolute number of osteoclasts (N.Oc) was estimated using the physical fractionator [29] on endosteal as well as periosteal bone surfaces as previously described in detail [21]. Briefly, the number of osteoclasts minus the number of bridges was divided with the area sampling fraction (asf) and the section sampling fraction (ssf). Finally, the result was divided by two because we counted both ways (i.e. both the appearance and the disappearance of osteoclasts).

### Coefficient of Error

The average coefficient of error (CE) for number estimation was evaluated using the formulas described earlier [21]. CE for surface estimates could not be calculated as the formula for determining the CE for a surface estimate was not valid due to the large size variation between different sections, when estimating the reference volume.

CE for the absolute osteoclast number (N.Oc) was 14.8% on endosteal surfaces and 12.2% on periosteal surfaces, which is considered acceptable.

### Statistics

Data was analyzed using STATA (version 11, Statacorp, College station, USA). Unpaired comparisons were conducted using the two sample Wilcoxon rank-sum test (Mann Whitney’s U test). Paired comparisons were conducted using the Wilcoxon signed-rank test. *P*-values less than 0.05 were considered statistically significant.

## Results

### Arthritis Score and Ankle Width

**Figure 2:**
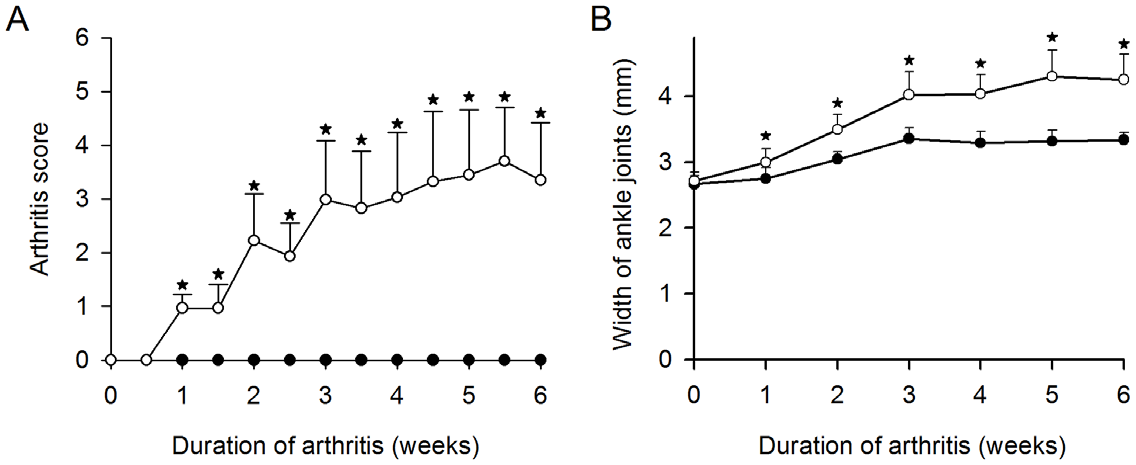
Comparison of female SKG mice with arthritis and female control mice. (**A**) Arthritis score in mice after arthritis induction with either mannan (open circles) or placebo (black circles). (**B**) Width of hind limb ankle joints after arthritis induction with either mannan (open circles) or placebo (black circles). Values are mean plus standard deviations. * indicates *p<*0.01. N = 10–11 mice per group.

The arthritis score was significantly higher in arthritic mice than in normal mice from week one and to the end of the study (Figure 2A). Likewise, the mean width of the hind limb ankle joints was significantly higher in the arthritic mice than in normal mice from week one and forward (Figure 2B).

### Bone Formation and Resorption on Endosteal and Periosteal Surfaces

**Figure 3:**
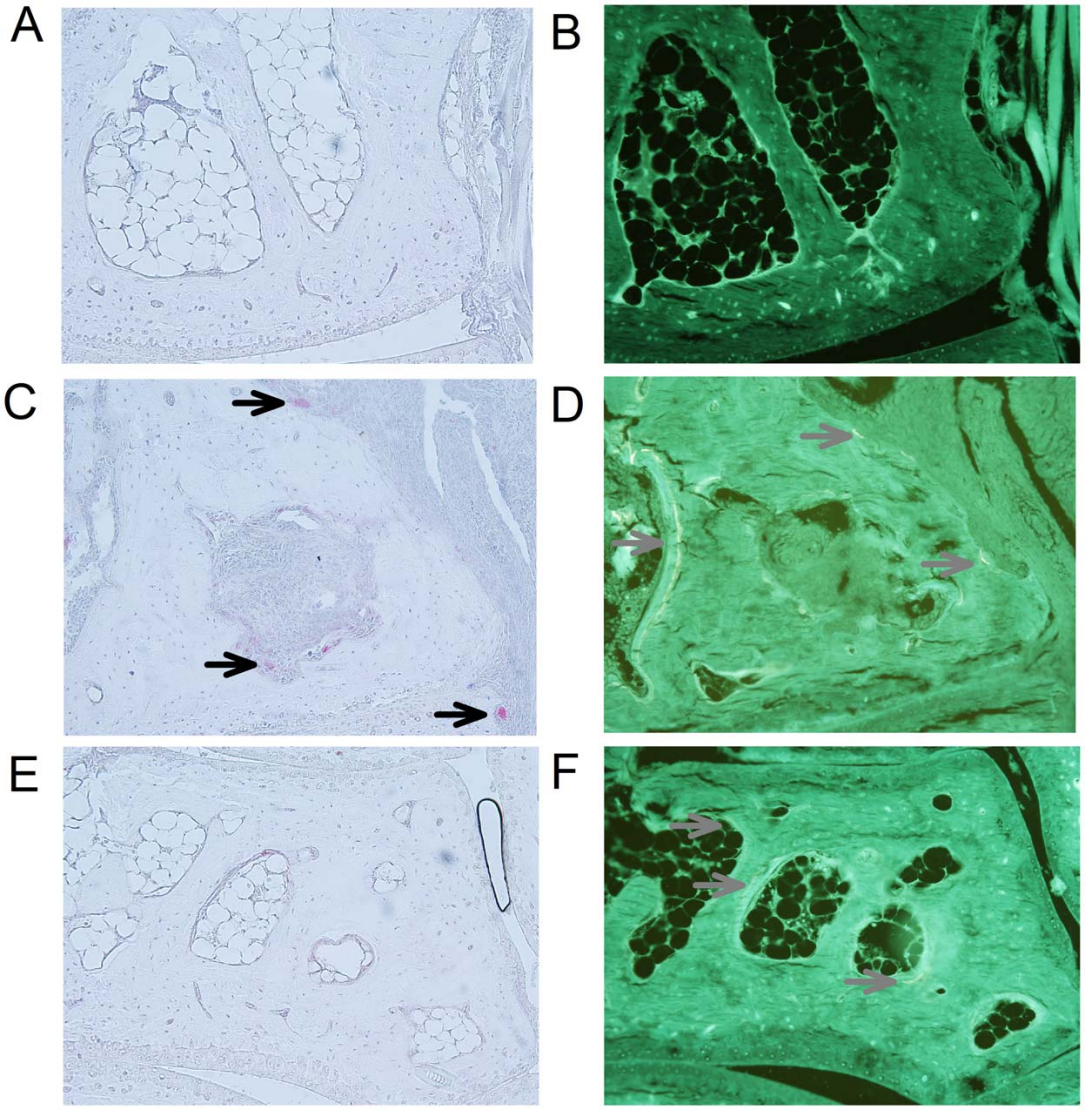
Histological pictures of bone resorption and formation in paws of SKG mice. (**A&B**) Histological sections 14 µm apart of the cuboid bone from a control mouse. No formation or resorption of bone is present. Bone resorption (**C**) and formation (**D**) in a mouse with arthritis and inflammation adjacent to the cuboid bone (sections are 28 µm apart). Oc.S/BS, ES/BS and MS/BS are increased compared with control animals. Bone resorption (**E**) and formation (**F**) in a mouse with arthritis but without inflammation adjacent to the navicular bone (sections are 14 µm apart). MS/BS is increased compared with control mice. Osteoclasts are marked with black arrows and mineralizing surfaces with grey arrows. A total magnification of ×457 was used.

**Figure 4:**
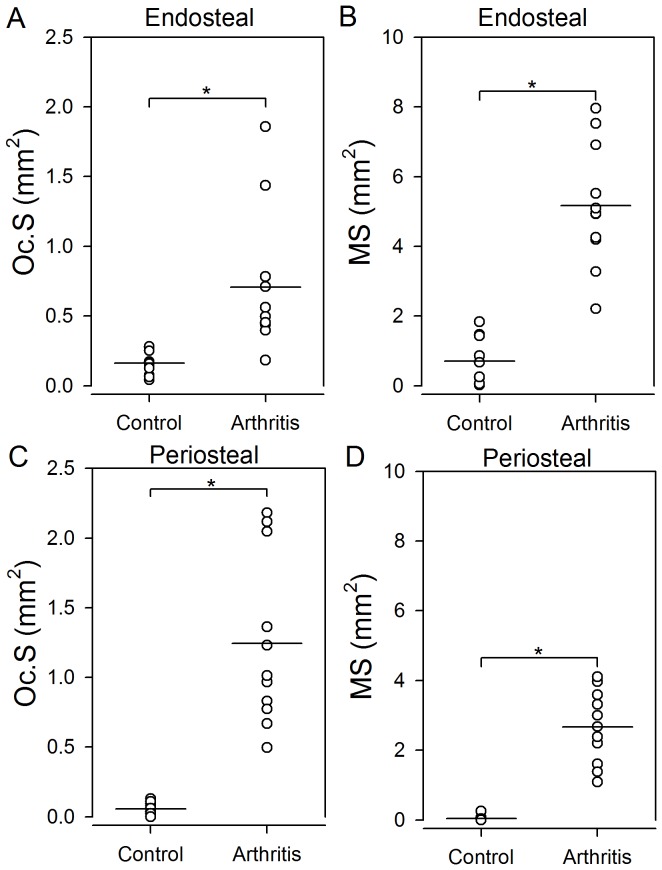
Surfaces with Bone formation and resorption were increased in mice with arthritis compared to control mice. Oc.S, and MS are shown on endosteal surfaces (**A–B**) and periosteal surfaces (**C–D**). Horizontal lines indicate mean values. * indicates *p*<0.001 comparing the two groups. N = 10–11 mice per group.

The resorptive bone surface area was larger in arthritic mice than in normal mice (Figure 3A&C). Arthritic mice had significantly larger ES and Oc.S on both endosteal and periosteal surfaces than normal mice (*p*<0.001). The results for ES and Oc.S were similar, and consequently only the findings for Oc.S are presented graphically (Figure 4A&C). Likewise, the formative bone surface area was larger in arthritic mice than in normal mice (Figure 3B&D). MS was significantly (*p*<0.001) larger in arthritic mice on both endosteal (Figure 4B) and periosteal (Figure 4D) surfaces than in normal mice. The relative values Oc.S/BS, ES/BS, and MS/BS on endosteal and periosteal surfaces were also statistically significantly higher in the arthritic mice than in the normal mice (data not shown). Finally, the results demonstrate that the ratio between Oc.S and MS was significantly larger on periosteal than on endosteal surfaces in arthritic mice (*p*<0.01) but not in control mice (*p = *0.27).

### Bone Formation on Surfaces with and without Inflammation in Arthritic Mice Compared to Normal Mice

**Figure 5:**
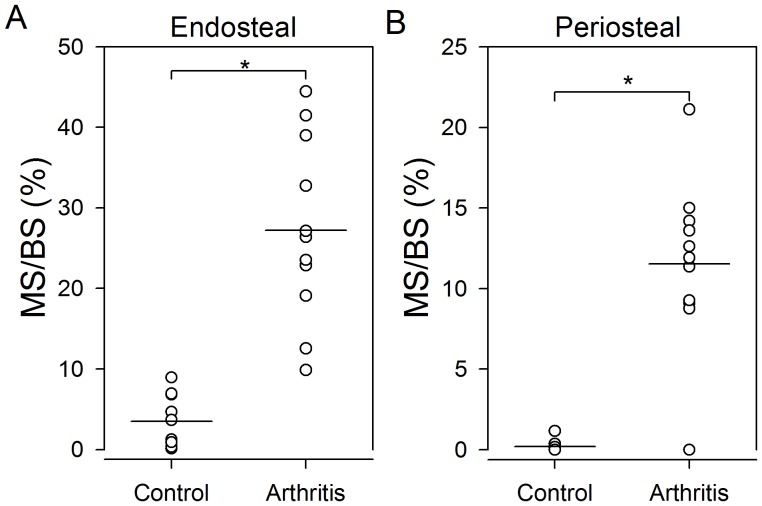
Surfaces with bone formation were increased adjacent to surfaces without inflammation in arthritic mice compared to bone surfaces in control mice. MS/BS on bone surfaces without inflammation shown on endosteal (**A**) and periosteal surfaces (**B**) in mice with arthritis and control mice. Horizontal lines indicate mean values. * indicates *p*<0.001. N = 10–11 mice per group.

The proportion of formative bone surfaces adjacent to non-inflamed tissue was higher in arthritic mice than in normal mice (Figure 3B&F). MS/BS adjacent to non-inflamed tissue was significantly (*p*<0.001) higher in arthritic mice than in normal mice on both the endosteal (Figure 5A) and the periosteal (Figure 5B) surfaces.

Likewise, MS/BS adjacent to inflamed tissue in arthritic mice was significantly higher than MS/BS in normal mice on both the endosteal and the periosteal surfaces (*p*<0.001).

### Bone Resorption and Formation on Surfaces Adjacent to Inflammation and Surfaces Adjacent to Normal Tissue in Arthritic Mice

**Figure 6:**
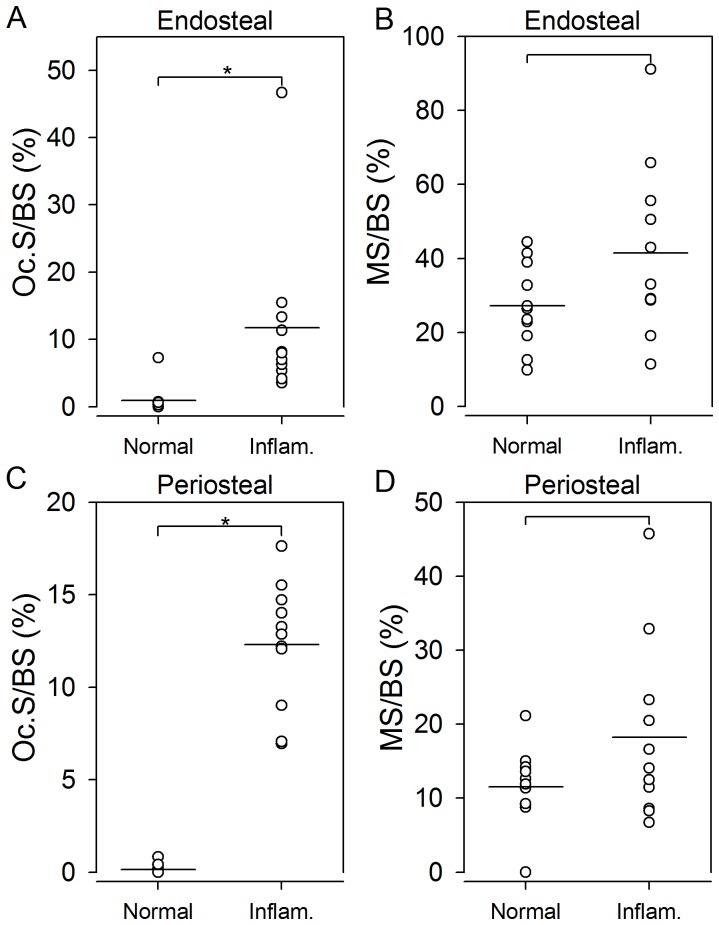
Surfaces with bone resorption but not bone formation were increased on surfaces adjacent to inflammation compared to surfaces adjacent to normal tissue in arthritic mice. In mice with arthritis Oc.S/BS were increased on surfaces adjacent to inflammation compared to normal surfaces, on both endosteal (**A**) and periosteal (**C**) surfaces. MS/BS was not different on surfaces adjacent to inflammation compared to normal surfaces on either endosteal or periosteal surfaces (**B&D**). Horizontal lines indicate mean values. * indicates *p*<0.01. N = 10–11 mice per group.

In arthritic mice, the proportion of resorptive bone surfaces was higher adjacent to inflammation than adjacent to tissue without inflammation (Figure 3C&E). Oc.S/BS and ES/BS were significantly higher on bone surfaces adjacent to inflammation than on bone surfaces adjacent to tissue without inflammation on both endosteal and periosteal surfaces (*p*<0.01). As seen above, the results for ES/BS and Oc.S/BS were similar, thus only the findings for Oc.S/BS are presented graphically (Figure 6A&C). In contrast, the proportion of formative bone surfaces were similar on bone surfaces adjacent to inflammation and bone surfaces adjacent to normal tissue in arthritic mice (Figure 3D&F). MS/BS did not differ between surfaces adjacent to inflammation and surfaces adjacent to normal tissue in arthritic mice on either endosteal (*p* = 0.155) or periosteal (*p* = 0.182) surfaces (Figure 6B&D).

### Osteoclast Number in Arthritic and Normal Mice

The absolute number of osteoclasts (mean±SD) were 5213±2792 in arthritic mice and 743±265 in normal mice (*p*<0.001) (Figure 3A&C). Likewise, the number of osteoclasts per bone surface was statistically significantly higher in arthritic mice than in normal mice.

## Discussion

It has been demonstrated that both osteoblastic bone formation and osteoclastic bone resorption are important for the bone loss seen in experimental arthritis [30]. However, present treatment modalities for RA targets inflammation and bone erosion but not bone formation [31]. Therefore, new insight into bone formation in arthritis is important for development of potential osteoblast targeting drugs for RA.

In the present study we showed that the area of formative bone surfaces as well as the area of resorptive bone surfaces was higher in arthritic mice than normal mice. In addition, the proportion of formative bone surfaces adjacent to tissue with and without inflammation was significantly higher in arthritic mice than in control mice. Moreover, the total number of osteoclasts was increased in arthritic mice compared to normal mice. Thus, both bone formation and bone erosion were elevated in arthritis. The ratio between Oc.S and MS were significantly different on the endosteal and periosteal surfaces in arthritis. Therefore, bone loss is probably not explained by an increased bone turnover alone. Instead, the activity of the osteoblasts may be partially inhibited compared to the osteoclasts. A lower activity of the osteoblasts compared to the osteoclasts may be caused by a down-regulation of the Wnt-pathway mediated through an elevation of TNF-α, which induces Dickkopf-1 expression [32]. In contrast to the findings of the present study a general down-regulation of bone formation in arthritis has previously been suggested [2].

We also investigated the impact of bone resorption in arthritis using sections stained for TRAP [33]. We found that the area of either erosive or osteoclast covered surfaces were very scarce in normal mice compared to arthritic mice. Furthermore, the proportion of surfaces with bone resorption was higher on bone surfaces adjacent to inflammation than on bone surfaces adjacent to tissue without inflammation in arthritic mice. Our results indicate that the inflammatory process is involved in the recruitment and activation of the osteoclast. This finding is not surprising as RANKL is increased in inflamed synovium of RA patients [34]. RANKL binds to its receptor, RANK on osteoclast precursors, thereby inducing osteoclast maturation and survival through TNF receptor-associated factor 6 (TRAF6) [3], [35], [36]. Likewise, macrophage colony-stimulating factor (M-CSF) is also increased in RA patients and in animal models of RA [37], [38].

We found that the proportion of formative bone surfaces did not differ between bone surfaces adjacent to normal or inflammatory tissue on either endosteal or periosteal surfaces in arthritic mice. The impact of local inflammation on bone formation on periosteal bone surfaces has not been investigated previously. However, on the endosteal surface, one research group found an elevation of the fraction of osteoblast-covered surfaces, the amount of osteoid, and mineralization labels adjacent to bone marrow inflammation [10]. Another research group demonstrated a decrease in MS/BS and bone formation rate (BFR/BS) adjacent to inflammatory bone marrow compared to surfaces adjacent to normal bone marrow [11]. There might be various reasons for these conflicting results. Surfaces undergoing active resorption cannot undergo formation at the same time. Therefore, bone with a relatively severe arthritis, might have a higher proportion of resorptive bone surfaces, leaving only a limited amount of bone surfaces available for bone formation. Other differences in the studies are the compartment investigated, the animal models used, and the fact that the histological sections may have been obtained at different stages of the disease in the various studies. Hence, future time course studies in different animal models are important in order to further clarify these issues.

Another important explanation for the conflicting findings may be the different evaluation methods applied. Traditional histomorphometry is a 2D model-based method, which means that assumptions about shape, size, orientation, and distribution of the cells or tissue are made. For example, cell profiles are counted in only a few sections. What happens in a situation where the number of cells are equal in the two groups, but the cells are bigger in one group? Then a larger number of cells will be estimated, because large cells are more likely to be sampled in a single section. In the present study we applied stereological 3D design based-methods avoiding assumptions about shape, size, orientation, and distribution for volume or number evaluation. For surface estimation the use of isotropic test lines is crucial, which we achieved by using the vertical sections design [26]. In addition, we used the principle of systematic, uniform random sampling [27]. In this method the sections are parallel and chosen at a fixed distance throughout the entire paw, but the location of the first section is selected randomly. In contrast, classical histomorphometry apply standardized sampling. Therefore, if the distribution is not uniform in the two groups, the localization where the sections are cut, can actually determine whether a difference between the groups is found. Consequently, stereology may be superior to the methods used in traditional histomorphometry.

### Conclusions

We demonstrated that in experimental autoimmune arthritis, the inflammation lead to an increase in resorptive bone surfaces as well as formative bone surfaces. The bone formation response may be more general, since formative bone surfaces were also increased when not associated with inflammation. Therefore, bone loss in arthritis may be the result of excessive local bone resorption, which cannot be compensated by the increased local bone formation. These findings may be valuable for the development of new osteoblast targeting drugs in RA.
